# 2516 Ultra-low Na18F tracer dosing for preclinical skeletal imaging enables new concepts in digital PET/CT

**DOI:** 10.1017/cts.2018.144

**Published:** 2018-11-21

**Authors:** Maria I. Menendez, Richard Moore, Katherine Binzel, Michael Friel, Jun Zhang, Rebecca Jackson, Michael Knopp

**Affiliations:** The Ohio State University

## Abstract

OBJECTIVES/SPECIFIC AIMS: The aim of this study was to assess the ultra-dose Na18F dPET protocol feasibility for skeleton imaging in a canine model with reduced radiation dose and preserved quantitative characteristics. We hypothesized that administering an ultra-low Na18F dose would provide suitable image quality while reducing subject’s exposure to radiation. METHODS/STUDY POPULATION: In total, 13 adult male beagles [weight (kg) mean±SD; 14.3±2.2] were scanned. The dogs were administered 3 different Na18F doses: 3 (standard dose/SD), 1 (low dose/LD), and 0.05 (ultra-low dose/ULD) mCi. Imaging started ≃45 minutes post injection for ≃ 33 minute total acquisition time. Covering the whole body, 11 bed positions, acquiring 120 (3 mCi) and 180 (1, 0.05 mCi) seconds per bed position. All imaging was performed on a digital photon counting system (Philips Vereos, pre-commercial release). PET list mode data were reconstructed using Time-of-flight with 4, 2, and 1 mm^3^ voxel volumes. Point spread function, and Gaussian filtering were applied. Two experienced blinded readers evaluated image sets overall quality, tissue characterization, and quality of background in the whole body skeleton. Three-dimensional (3D) regions of interest (ROI) were traced over the distal femur, first lumbar vertebra, and a portion of the liver, recording standard uptake values (SUVmax and SUVmean). RESULTS/ANTICIPATED RESULTS: All the scans and reconstructions were successfully completed in all subjects. Decreasing Na18F dose from the standard dose (3 mCi) to the ultra-low dose/ULDO (0.05 mCi), demonstrated acceptable image quality and quantification. Ultra-low dose Na18F SUVmean values for the 3D ROIs reported (mean±SD) 2.6±0.7, 2.5±1.1, 9±1.6, and 0.6±0.3 from the right and left distal femur, first lumbar vertebra, and a portion of the liver, respectively. When compared the SD with the LD and ULD, dPET demonstrated acceptable image quality and definition for qualitative overall assessment. This was also found for the overall quantitative ROI assessment of the healthy canine skeletons. DISCUSSION/SIGNIFICANCE OF IMPACT: Ultra-low dose Na18F at a level of 50 μCi for a 14 kg canine appears to be diagnostically feasible and a robust option to reduce (60-fold) radiotracer doses in a translational animal model using a dPET system. Furthermore, it allows us to move preclinical nuclear medicine imaging forward with substantial reduced exposure levels while preserving image quality. Both visual and quantitative results indicate that the standard-dose bone Na18F dPET can be decreased with a satisfactory diagnostic image quality. Ultra-low Na18F dose is indeed important for younger populations, control patients, and nononcological diseases/conditions. Favorable pharmacokinetics of Na18F (such as high bone uptake, minimal binding to serum proteins, rapid single-pass extraction, and fast clearance from the soft tissues) in addition to the technological capabilities of dPET/CT demonstrated feasibility enabling dose reduction strategies. Ultra-low dose has diagnostic reproducibility and lower radiation burden compared with higher fixed dose techniques in current available guidelines [Society of Nuclear Medicine and Molecular Imaging; SNMMI (5–10 mCi)]. Na18F dPET/CT provides higher sensitivity and diagnostic accuracy, which enables high-quality images with lower tracer activity in this translational animal model. Future research will apply the same methodology to other anatomical targets as well as to the use of different tracers. Preclinical nuclear medicine imaging using ultra-low tracer doses, demonstrated the potential to obtain reasonable quality images and diminishing radiation surveillance in accordance with as low as reasonably achievable tracer levels.

